# Renal cell carcinoma with melanin pigment

**DOI:** 10.4103/0970-1591.65407

**Published:** 2010

**Authors:** Jayaprakash Shetty, Chandrika, Prabhu Laxman

**Affiliations:** Department of Pathology, K. S. Hegde Medical Academy, Deralakatte, India; 1 Unity Health Complex, Mangalore, India

**Keywords:** Melanin, pigment, renal cell carcinoma

## Abstract

The incidence of renal cell carcinoma has been steadily increasing. There are several morphological types of renal cell carcinoma. Recognizing histologic patterns of renal cell carcinoma is important for correct diagnosis and subsequent medical care for the patient. Melanotic tumors in the kidney are very rare. Here, we present an unusual case of renal cell carcinoma with melanin pigment.

## INTRODUCTION

Renal cell carcinoma accounts for approximately 3% of adult malignancies and 90%-95% of neoplasms arising from the kidney.[1] The age-adjusted incidence of renal cell carcinoma is increasing by 3% per year.

Renal cell carcinoma has five histologic subtypes: clear cell (75%), chromophilic (15%), chromophobic (5%), oncocytoma (3%) and collecting duct (2%).[2] Melanotic tumors in the kidney are extremely rare. Pigments other than hemosiderin can appear in different types of renal cell carcinoma. This feature expanded the spectrum of histologic features of renal cell carcinoma and at the same time difficulty in diagnosing the tumor.

Two cases of renal cell carcinoma with melanin pigment have been reported so far. In one case, the pigment was thought to be an accumulation of abnormal lysosomal granules[3] and another was a case of pigmented renal cell carcinoma showing melanocytic differentiation proved with Masson Fontana and HMB–45 stains.[1] Here, we present a similar case of renal cell carcinoma with melanin pigment.

## CASE REPORT

A 20-year-old female patient presented with complaints of pain in the abdomen in February 2008, with no other symptoms. Abdomen examination revealed a soft tender mass measuring 4 cm in the right lumbar region. No other clinical abnormalities were detected. Ultrasound abdomen showed a mass lesion in the right lumbar region. In February 2008, the patient underwent radical nephrectomy. The pathological diagnosis of pigmented renal cell carcinoma was made.

**Figure 1:**
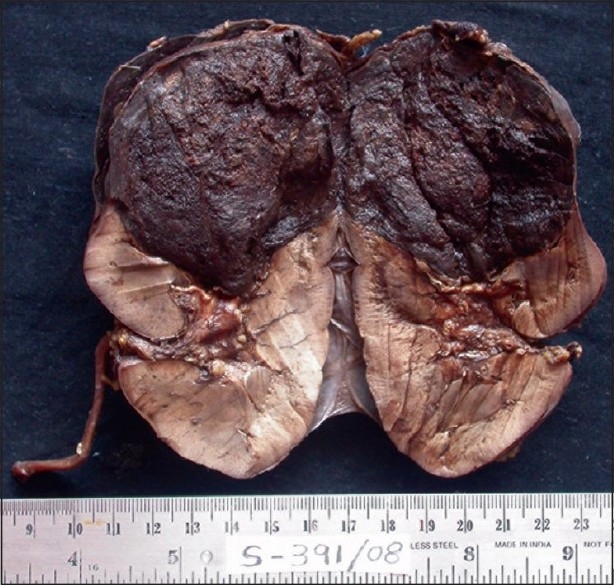
Gross appearance of the tumor. Tumor replaces the parenchyma of the upper pole of the kidney and consists of a solid multilobular dark brown tumor.

Pathological findings – The surgical radical nephrectomy specimen consisted of right kidney measuring 13 × 7 × 6.5 cm, ureter measuring 6.5 cm in length. Cross-section showed dark brown to tan tumor in the upper pole measuring 5 cm in diameter. Tumor was multilobulated and solid. The remaining renal parenchyma appeared normal including pelvicalyceal system [Figure 1]

Microscopically, the kidney tumor was predominantly composed of clear cells with focal granular cells. Tumor cells were arranged in acinar, solid and tubular patterns.

**Figure 2:**
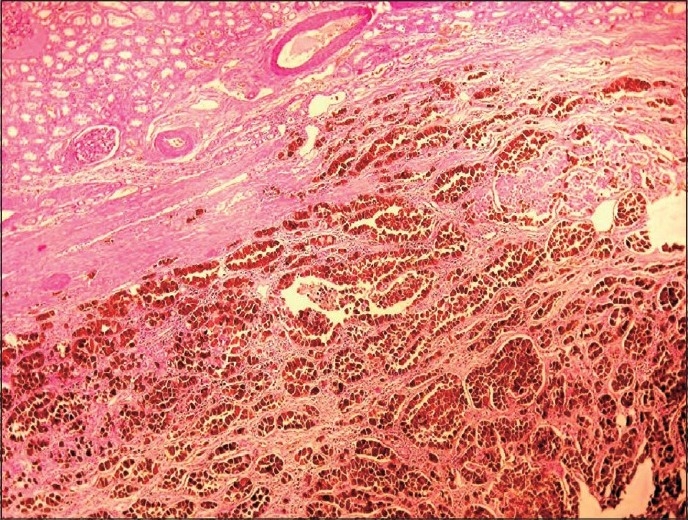
Tumor cells showing dark brown pigment in the cytoplasm (H and E, ×10)

The tumor cells had clear cytoplasm, lacked significant pleomorphism and had low Fuhrman nuclear grade (II/IV). Tumor cells were separated by highly vascular and fibrous stroma. In the majority of tumor cells, dark brown pigment was observed in the cytoplasm of clear cells. The pigment was dark brown, granular, nonrefractile [Figure 2].

Tumor capsule was free of tumor tissue. The renal vessels and ureter were free of disease. Special stain was performed on formalin-fixed and paraffin-embedded material. The pigment showed strongly black stain with Fontana Masson.

## DISCUSSION

Renal cell carcinoma develops in the lining of the tubules. The new classification is based on morphology, histochemistry, clinical behavior and genetic alterations, consisting of clear cell, chromophilic, chromophobic, oncocytic and collecting duct.[2] The first three types are most frequently seen and constitute 95% of renal cell carcinoma. These subtypes are distinct, which has been proved by cytogenetics and molecular studies.[1,2,4]

Clear cell carcinoma accounts for 75% of renal cell carcinoma. Cells are unusually clear with cytoplasm rich in glycogen and lipids. Sometimes cytoplasm is eosinophilic. This tumor may be cystic or solid. Growth pattern is usually acinar but occasionally of sarcomatoid or tubulopapillary architecture. Clear cell carcinoma is characterized by chromosome 3p deletion.[1,3] Chromophilic carcinoma in which most cases classified previously as tubulopapillary, comprises approximately 14% of renal cell carcinoma are often bilateral, multifocal, may have trisomy 7 and/or trisomy 17 and have a better 5-year survival.[1,2] Chromophobic carcinoma comprises approximately 5% of renal cell carcinoma; it is characterized by a hypodiploid chromosome number and multiple chromosome losses but does not exhibit the 3p deletion.[2] Patients with these tumor have favorable prognosis.

The endogenous pigments other than hemosiderin have been observed in renal cell carcinoma. It was first reported in 1995.[1,3] A total of 11 cases have been reported in literature. It is important to recognize the presence of these pigments in renal cell carcinoma and differentiate it from metastatic malignant melanoma.[1,3] It is an important differential diagnosis when we consider various metastatic deposits in the kidney and requires stringent follow-up. Pigment in our case is composed of dark brown nonreflectile fine granules and stained black with Masson Fontana, consistent with melanin. It has been proposed that pigmented renal cell carcinoma may be a unique variant of renal cell carcinoma.[1,5] The significance of pigment in renal cell carcinoma other than differential diagnosis is yet to be established.

Dark brown endogenous pigments other than melanin found in renal cell carcinoma are hemosiderin, homogentisic acid and lipofuschin. Hemosiderin granules are variably sized, reflective and golden brown and can be confirmed with Perl's stain.

Lipofuschin shows perinuclear location. Homogentisic acid is a brown black pigment with alkaptonuria, a rare metabolic disease. In summary, we report a pigmented clear cell renal carcinoma. The pigments are in the form of fine granules and nonreflectile and staining was performed using Fontanna Masson.

This feature expands the spectrum of morphological changes and information about differential diagnosis of renal cell carcinoma.
